# Supplementary material for: Modeling glioblastoma heterogeneity as a dynamic network of cell states
Source: Mol Syst Biol. 2021 Sep 16;17(9):e10105. doi: 10.15252/msb.202010105 (PMC8444284; doi:10.15252/msb.202010105)
Supplement: Supplementary file 6 — Source Data for Figure 5 [file MSB-17-e10105-s004.zip › Figure5A_sourcedata/GSEA_3071/nosigdb_stateIII.GseaPreranked.1622795155562/NB_KI_CLUSTER18.html]

Details for gene set NB\_KI\_CLUSTER18[GSEA]

|  || Dataset | state33071 |
| Phenotype | NoPhenotypeAvailable |
| Upregulated in class | na\_neg |
| GeneSet | NB\_KI\_CLUSTER18 |
| Enrichment Score (ES) | -0.64019585 |
| Normalized Enrichment Score (NES) | -3.4700987 |
| Nominal p-value | 0.0 |
| FDR q-value | 0.0 |
| FWER p-Value | 0.0 |
Table: GSEA Results Summary

  

Fig 1: Enrichment plot: NB\_KI\_CLUSTER18      
 Profile of the Running ES Score & Positions of GeneSet Members on the Rank Ordered List

  

| PROBE | GENE SYMBOL | GENE\_TITLE | RANK IN GENE LIST | RANK METRIC SCORE | RUNNING ES | CORE ENRICHMENT || 1 | MT2A |  |  | 31 | 0.499 | 0.0078 | No |
| 2 | LGALS1 |  |  | 230 | 0.323 | -0.0330 | No |
| 3 | CYTOR |  |  | 258 | 0.306 | -0.0302 | No |
| 4 | MYL9 |  |  | 293 | 0.293 | -0.0297 | No |
| 5 | TPM4 |  |  | 306 | 0.290 | -0.0236 | No |
| 6 | TAGLN |  |  | 407 | 0.257 | -0.0413 | No |
| 7 | FILIP1L |  |  | 454 | -0.252 | -0.0451 | No |
| 8 | THY1 |  |  | 472 | -0.253 | -0.0415 | No |
| 9 | MYLK |  |  | 479 | -0.253 | -0.0350 | No |
| 10 | PHLDA1 |  |  | 589 | -0.259 | -0.0549 | No |
| 11 | FCGRT |  |  | 713 | -0.265 | -0.0782 | No |
| 12 | MFGE8 |  |  | 794 | -0.269 | -0.0903 | No |
| 13 | ID3 |  |  | 880 | -0.273 | -0.1036 | No |
| 14 | TFPI |  |  | 1141 | -0.287 | -0.1616 | No |
| 15 | COL18A1 |  |  | 1302 | -0.295 | -0.1935 | No |
| 16 | CDKN1A |  |  | 1631 | -0.314 | -0.2681 | No |
| 17 | CEBPD |  |  | 1819 | -0.325 | -0.3060 | No |
| 18 | TIMP1 |  |  | 2038 | -0.337 | -0.3516 | No |
| 19 | SERTAD1 |  |  | 2055 | -0.338 | -0.3450 | No |
| 20 | SERPINF1 |  |  | 2245 | -0.352 | -0.3825 | No |
| 21 | ADAMTS1 |  |  | 2736 | -0.394 | -0.4964 | No |
| 22 | NOTCH3 |  |  | 2759 | -0.395 | -0.4896 | No |
| 23 | SERPINH1 |  |  | 2921 | -0.410 | -0.5181 | No |
| 24 | CD63 |  |  | 3258 | -0.452 | -0.5904 | No |
| 25 | C1R |  |  | 3364 | -0.473 | -0.6025 | No |
| 26 | ZFP36L1 |  |  | 3487 | -0.503 | -0.6180 | No |
| 27 | ITGB1 |  |  | 3574 | -0.537 | -0.6232 | Yes |
| 28 | RRBP1 |  |  | 3595 | -0.545 | -0.6110 | Yes |
| 29 | IGFBP7 |  |  | 3611 | -0.554 | -0.5973 | Yes |
| 30 | RGS16 |  |  | 3616 | -0.555 | -0.5808 | Yes |
| 31 | FOS |  |  | 3621 | -0.561 | -0.5640 | Yes |
| 32 | PRSS23 |  |  | 3658 | -0.584 | -0.5548 | Yes |
| 33 | COL4A2 |  |  | 3664 | -0.586 | -0.5375 | Yes |
| 34 | COL6A2 |  |  | 3674 | -0.595 | -0.5209 | Yes |
| 35 | PDGFRB |  |  | 3678 | -0.598 | -0.5027 | Yes |
| 36 | TIMP3 |  |  | 3755 | -0.677 | -0.5009 | Yes |
| 37 | LIMA1 |  |  | 3774 | -0.706 | -0.4831 | Yes |
| 38 | TGFBI |  |  | 3781 | -0.713 | -0.4621 | Yes |
| 39 | SPARC |  |  | 3811 | -0.759 | -0.4455 | Yes |
| 40 | BGN |  |  | 3812 | -0.761 | -0.4214 | Yes |
| 41 | LHFPL6 |  |  | 3814 | -0.762 | -0.3975 | Yes |
| 42 | COL4A1 |  |  | 3829 | -0.791 | -0.3760 | Yes |
| 43 | FN1 |  |  | 3850 | -0.842 | -0.3544 | Yes |
| 44 | COL6A1 |  |  | 3863 | -0.872 | -0.3299 | Yes |
| 45 | NEAT1 |  |  | 3880 | -1.005 | -0.3022 | Yes |
| 46 | CYR61 |  |  | 3887 | -1.037 | -0.2708 | Yes |
| 47 | A2M |  |  | 3890 | -1.053 | -0.2380 | Yes |
| 48 | IGFBP5 |  |  | 3895 | -1.082 | -0.2047 | Yes |
| 49 | COL6A3 |  |  | 3900 | -1.171 | -0.1686 | Yes |
| 50 | COL1A1 |  |  | 3902 | -1.191 | -0.1311 | Yes |
| 51 | COL1A2 |  |  | 3906 | -1.278 | -0.0913 | Yes |
| 52 | COL3A1 |  |  | 3918 | -1.418 | -0.0492 | Yes |
| 53 | MGP |  |  | 3927 | -1.633 | 0.0005 | Yes |
Table: GSEA details [plain text format]

  

Fig 2: NB\_KI\_CLUSTER18: Random ES distribution      
 Gene set null distribution of ES for **NB\_KI\_CLUSTER18**
